# Thymol as a Component of Chitosan Systems—Several New Applications in Medicine: A Comprehensive Review

**DOI:** 10.3390/plants13030362

**Published:** 2024-01-25

**Authors:** Adam Kowalczyk, Bartosz Twarowski, Izabela Fecka, Carlo Ignazio Giovanni Tuberoso, Igor Jerković

**Affiliations:** 1 Department of Pharmacognosy and Herbal Medicines, Faculty of Pharmacy, Wroclaw Medical University, 50-556 Wroclaw, Poland; bartosz.twarowski@student.umed.wroc.pl (B.T.); izabela.fecka@umw.edu.pl (I.F.); 2 Department of Life and Environmental Sciences, University of Cagliari, University Campus, S.P. Monserrato-Sestu Km 0.700, 09042 Monserrato, CA, Italy; tuberoso@unica.it; 3 Department of Organic Chemistry, Faculty of Chemistry and Technology, University of Split, 21000 Split, Croatia

**Keywords:** thymol, chitosan, combined products, antimicrobial materials, drug delivery

## Abstract

Thymol, a plant-derived monoterpene phenol known for its broad biological activity, has often been incorporated into chitosan-based biomaterials to enhance therapeutic efficacy. Using the Preferred Reporting Items for Systematic Reviews and Meta-Analysis (PRISMA) guidelines, we conducted a systematic literature review from 2018 to 2023, focusing on the biomedical implications of thymol-loaded chitosan systems. A review of databases, including PubMed, Scopus, and Web of Science was conducted using specific keywords and search criteria. Of the 90 articles, 12 were selected for the review. Thymol-loaded chitosan-based nanogels (TLCBS) showed improved antimicrobial properties, especially against multidrug-resistant bacterial antagonists. Innovations such as bipolymer nanocarriers and thymol impregnated with photosensitive chitosan micelles offer advanced bactericidal strategies and show potential for bone tissue regeneration and wound healing. The incorporation of thymol also improved drug delivery efficiency and biomechanical strength, especially when combined with poly(dimethylsiloxane) in chitosan–gelatin films. Thymol–chitosan combinations have also shown promising applications in oral delivery and periodontal treatment. This review highlights the synergy between thymol and chitosan in these products, which greatly enhances their therapeutic efficacy and highlights the novel use of essential oil components. It also highlights the novelty of the studies conducted, as well as their limitations and possible directions for the development of integrated substances of plant and animal origin in modern and advanced medical applications.

## 1. Introduction

Functional biomaterials (FBs) are designed to exhibit specific properties [1]. Owing to their biocompatibility, biodegradability, and hydrophilicity, they are used in many areas of life, as well as in medicine for therapeutic applications [2,3,4,5,6]. They can be of natural origin, synthetic, or composite forms [7,8].

The application of FBs in living tissues involves many steps, such as surface modification, covalent coupling, or the integration of specific ligands to improve cell adhesion, tissue differentiation and regeneration [1,9]. Such interactions are of great importance in medical applications that promote the mechanical support, growth, and delivery of therapeutic agents [1,10,11].

**Figure 1:**
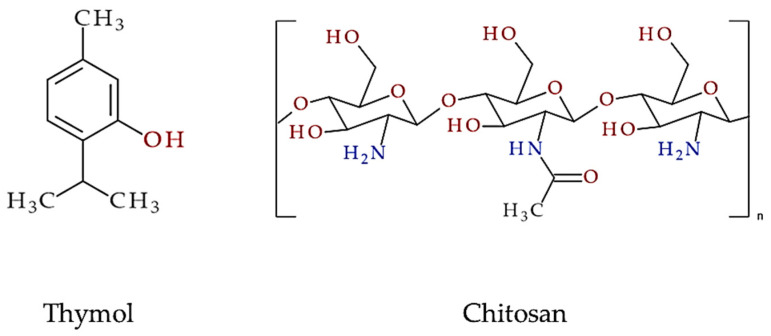
Structural formula of thymol and chitosan.

Chitosan (Figure 1), classified as a polysaccharide formed from chitin through partial chemical deacetylation (composed of randomly distributed β-(1→4)-linked D-glucosamines and *N*-acetyl-D-glucosamines), exhibits a wide range of biological activities and has bacteriostatic, antioxidant, anti-inflammatory effects [12,13]. Biodegradability, biocompatibility, low toxicity, and adhesive properties are some of the beneficial properties of FB with chitosan [14,15]. Furthermore, the use of chitosan in biomedical materials is highly favored because of its widespread availability and minimal negative effects on the environment and human population. Therefore, chitosan-derived materials have been widely studied and used in medicine as surgical or wound dressing materials [16,17].

Thymol (5-methyl-2-(propan-2-yl)phenol or *p*-cymene-3-ol; Figure 1) is a natural monoterpenoid phenol derivative of *p*-cymene found in many botanical families (e.g., Lamiaceae, in the genera of *Thymus*, *Origanum*, *Satureja*, *Monarda*) and has a variety of biological applications, mainly focusing on expectorant, antibacterial, antifungal, antiviral, antioxidant, and anti-inflammatory activities [18,19]. It acts as a biocide in pharmaceuticals, cosmetics, and food products by disrupting bacterial membranes. Together with carvacrol, thymol is the main constituent of thyme volatile oil (*Thymi typo thymolo aetheroleum*). It can also be obtained by the chemical synthesis of aromatic hydrocarbons such as *p*-cymene or the alkylation of *m*-cresol. Thyme oil and thymol are used therapeutically for respiratory infections and the inflammation of mucous membranes and skin. It is administered as an oral, inhaled, or topical formulation.

Studies have shown that the addition of thymol increases the elasticity of chitosan-based wound dressings [20]. However, the addition of thymol can reduce the tensile strength of other materials [21].

The interactions between thymol–chitosan biomaterials (CTBs) and living tissues involve both cellular and molecular mechanisms. At the cellular level, chitosan-based materials promote the growth of fibroblasts and capillaries, which are essential for wound healing, for example [22]. They also modulate the proliferative capacity, growth, and polarization behavior of macrophages [23]. Thymol–chitosan hydrogels were found to be biocompatible after exposure to fibroblasts and showed antimicrobial activity against *Staphylococcus aureus* and *Streptococcus mutans* [24]. Chitosan has antimicrobial properties that promote human cell proliferation and tissue repair [25].

Although CTBs offer promising benefits, their limitations should be noted. One of the main challenges and limitations to consider in CTBs is the poor solubility of chitosan in neutral and basic media, which limits their use under certain conditions [26]. This could potentially limit their application in environments with varying pH levels. While chitosan is generally considered biocompatible with living tissue, there may be issues related to the body’s response to these materials, which may affect their effectiveness [27].

This review highlights the growing potential of thymol, a compound found in essential oils, which, in combination with animal-derived chitosan, opens up new perspectives in innovative, applied and interdisciplinary medical research due to the research methods and the main results obtained. This article aims to summarize the key findings and limitations of the presented results and identify future research directions. It also aims to provide information on the multifaceted potential of thymol–chitosan nanogels in addressing various healthcare challenges. Table 1 provides a general overview of the research topics, synopsis, and results included in the articles related to TLCBS.

This review article follows the PRISMA guidelines to provide a comprehensive and transparent approach to collecting and analyzing the relevant literature on the current state of knowledge of thymol as a component of chitosan systems.

Research question: The research question for this review is: “What is the current state of knowledge on the biomedical implications of thymol-loaded chitosan systems?”

Literature search: A systematic literature search was conducted to identify relevant studies published in English between 2018 and 2023. The following electronic databases were searched: PubMed, Scopus, and the Web of Science. The keywords used included combinations of keywords such as ‘thymol’, ‘chitosan, ‘biomedical applications’, ‘drug delivery’ and ‘antimicrobial materials.’

Study selection: The article selection process was carried out in two stages: prescreening titles and abstracts, followed by full-text evaluation. Two independent reviewers analyzed the titles and abstracts of the retrieved articles to identify potentially relevant reports. The full texts of potentially eligible articles were retrieved and assessed against the predefined inclusion and exclusion criteria.

Inclusion criteria:

- Studies on the use of thymol as an ingredient in chitosan-based functional biomaterials.
- Research published in English between 2018 and 2023.
- Research on biomedical applications, such as drug delivery and antimicrobial materials.

Exclusion criteria:

- Research did not focus on thymol-based biomaterials.
- Studies published before 2018 or in languages other than English.
- Studies not related to biomedical applications.

Data extraction: Data extraction was independently performed by two reviewers. The following information was extracted from each study: authors, year of publication, objectives of the study, study design, biomaterial synthesis methods, characterization techniques, application areas, main findings, and conclusions.

Data synthesis and analysis: The extracted data were synthesized and analyzed to provide a comprehensive overview of the current state of knowledge regarding thymol as a component of chitosan functional biomaterials. The results of the studies were categorized and presented according to their areas of application, such as antimicrobial materials and drug delivery.

**Figure 2:**
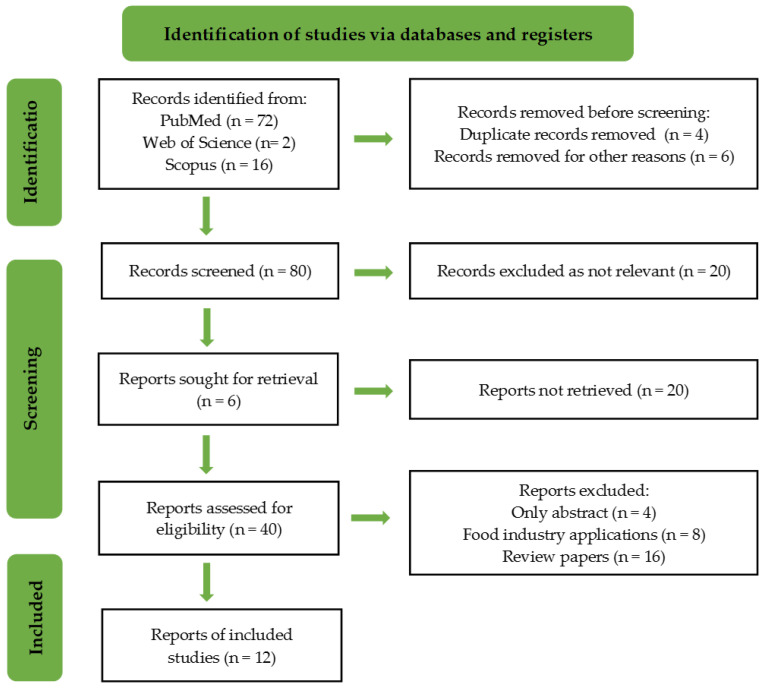
PRISMA flowchart of the included studies.

The PRISMA flow chart of the included studies is shown in Figure 2.

## 2. Thymol and Chitosan in Nanogel Formulations

Nanogels have emerged as versatile drug delivery systems that offer the controlled release, enhanced bioavailability, and targeted delivery of therapeutic agents. Among the various bioactive compounds, thymol is incorporated into these carriers to exploit its potent antimicrobial and anti-inflammatory properties. Piri-Gharaghie et al. [28] investigated nanogels, particularly those based on chitosan, as thymol delivery carriers. Thymol, in a concentration of 0.25 mg/mL in ethanol, was encapsulated in a chitosan nanogel as a novel pharmaceutical formulation and evaluated for its encapsulation efficiency, in vitro drug release, and stability. The particle size, zeta potential, and morphology of the thymol-encapsulated chitosan nanogels (Ty-CsNGs) were also evaluated. Ty-CsNG was tested against multidrug-resistant bacterial strains, including *Staphylococcus aureus*, *Acinetobacter baumannii* and *Pseudomonas aeruginosa*. Ty-CsNG showed a significant reduction in the minimum inhibitory concentration (MIC), outperforming free thymol by 4–6 times. Thus, against *Acinetobacter baumannii*: thymol: 128–512 μg/mL, Ty-CsNG: 32–128 μg/mL; against *S. aureus*: thymol: 16–128 μg/mL, Ty-CsNG: 8–64 μg/mL; and against *P. aeruginosa*: thymol: 1–32 μg/mL, Ty-CsNG: 2–32 μg/mL. The smaller size of Ty-CsNG (82.71 ± 9.6 nm) compared to free thymol may have facilitated its interaction with bacterial cells, leading to increased antimicrobial activity. Ty-CsNG also reduced the expression of ompA and pgaB in all strains. This could be due to the interaction of thymol and transcription factors, which are deactivated; biofilm genes not being transcribed; and biofilm genes being reduced in expression. Ty-CsNG exhibited 60 days’ stability at 4 °C, with an average size of 82.71 nm and an encapsulation efficiency of 76.54%. Sreelatha et al. [40] also confirmed that thymol-loaded chitosan nanoparticles are potent antimicrobials against another pathogen, *Xanthomonas campestris pv. campestris*. These formulations exhibited significant antibacterial activity, with MICs ranging from 100 to 600 μg/mL.

The novelty of the study by Piri-Gharaghie et al. is the development of a new formulation of Ty-CsNG as a potential antimicrobial agent. Research on the antibiofilm mechanism of Ty-CsNG suggests that one of its mechanisms is to reduce the number of microorganisms and subsequent biofilm formation. This ability—to penetrate the biofilm structure more easily due to binding to chitosan—represents a new aspect of the antimicrobial activity of the nanogel. This study also provides evidence of a stronger inhibitory effect of the chitosan nanogel against Gram-negative bacteria, providing a new report that contributes to the understanding of the differential antimicrobial activity of chitosan-based formulations against different types of bacteria.

## 3. Tragacanth/Chitosan Nanocarriers for Efficient Thymol Delivery

In the field of drug delivery, the use of bipolymer nanocarriers has increased exponentially, owing to their potential to offer enhanced stability, release profiles, and better biocompatibility. To optimize the therapeutic efficacy and expand the use of thymol, its encapsulation in bipolymer nanocarriers has been investigated. In 2019, Sheorain et al. [29] used a mixture of tragacanth gum and chitosan as a nanocarrier to enhance the therapeutic properties of thymol. A thymol nanoformulation was prepared using an ionic complexation method in which thymol solution (4 mg/mL in ethanol) was added dropwise to tragacanth gum solution (20 mg/mL) and then to chitosan solution (5–20 mg/mL prepared in 1% (*v*/*v*) acetic acid) to produce ionically cross-linked nanoparticles. The best results were obtained at a concentration of 200 mg of tragacanth gum and 400 mg of chitosan with an encapsulation efficiency of 98.72%. It was synthesized in an aqueous medium via ionic complexation without a stabilizer. The optimized nanoformulation was confirmed using techniques such as transmission electron microscopy, atomic force microscopy, and Fourier-transform infrared spectroscopy. The authors found that the optimized nanoparticles had a spherical shape with a size range of 150–200 nm. At pH 7.4, the Korsmeyer–Peppas mathematical model, which describes the mechanism of drug release from the nanoparticle system, best expressed the release kinetics of thymol, which followed biphasic release kinetics with a rapid dissolution phase followed by a maintenance phase. The authors attributed the initial rapid dissolution phase to the physical adsorption of thymol in the nanoparticles, followed by a sustained-release phase of thymol bound to the core of the nanoparticles. The mechanism of thymol release from the nanoparticles followed the quasi-Fickian diffusion type, indicating controlled and sustained release. The human red blood cell (HRBC) stabilization method was used to evaluate the in vitro anti-inflammatory activity of the prepared nanoformulation. The results showed that all the tested samples protected against hypotonicity-induced hemolysis. The percent membrane stabilization was 91.07% for diclofenac sodium (0.2 mg/mL), 84.11% for thymol (0.5 mg/mL) and 89.60% for thymol-loaded NPs with a thymol concentration of 0.5 mg/mL. From these results, it is clear that thymol nanoparticles show better anti-inflammatory potential than pure thymol, which has been confirmed by previous studies [41]. In vitro, antioxidant activity evaluated by the DPPH method showed that the radical scavenging activity of thymol nanoparticles was significantly better than that of pure thymol and increased with an increasing thymol concentration (from 70% at a thymol concentration of 0.0625 mg/mL to 90% at a concentration of 0.5 mg/mL). For pure thymol, at the same concentrations, the results were 50% and 80%, respectively. Such a nanoformulation not only effectively encapsulated thymol but also enhanced its anti-inflammatory and antioxidant properties.

Sheorian et al. introduce a novel approach to the preparation of thymol nanoformulations using a tragacanth gum/chitosan nanocarrier to address thymol limitations, such as poor solubility and low susceptibility to oxidation. In addition, the evaluation of the antioxidant and anti-inflammatory potential of nanoparticles using the DPPH assay and HRBC membrane-stabilization methodology provides new insights into their efficacy.

## 4. Light-Controlled Chitosan Micelles for Thymol Delivery

The innovative combination of photonics and nanomedicine has given rise to the concept of light-controlled drug delivery systems that enable the spatial and temporal regulation of drug release. Micelles, with their core-shell structure, provide an ideal platform for encapsulating hydrophobic agents such as thymol. Combined with light-sensitive functionalities, chitosan micelles can provide a dynamic response to external light stimuli. A study on light-controlled chitosan micelles tailored to deliver thymol and its implications was conducted by Wang et al. [30]. using a groundbreaking approach. The authors reported the formation of light-controlled chitosan micelles impregnated with thymol, prepared via self-assembly using an amphiphilic copolymer consisting of toluidine blue O (TBO), grafted chitosan (CHI-TBO), and poly(propylene sulfide) (PPS) (TBO-CHI-PPS), which were specifically targeted to degrade bacterial biofilms. A notable feature of these micelles is their ability to generate reactive oxygen species (ROS) after irradiation. This ROS generation, combined with the release of thymol, led to enhanced bactericidal activity, which proved particularly destructive against *Listeria monocytogenes* and *S. aureus* biofilms. The amount of thymol used in the solution for micelles preparation was 1 mg in 1 mL of DMSO. For both *L. monocytogenes* and *S. aureus*, micelles impregnated with thymol at a concentration of 330 µg/mL showed a better biofilm-destroying capacity than thymol and micelles without thymol in the dark. The relative biofilm value for *L. monocytogenes* for pure thymol and for the formulation in the dark was 0.6; for *S. aureus, it* was 0.6 and 0.4, respectively. The MIC and minimum bactericidal concentration (MBC) values of TBO-CHI-PPS micelles against *S. aureus* were found to be 165 μg/mL and 330 μg/mL, respectively, and against *P. aeruginosa,* they were 330 μg/mL and 660 μg/mL, respectively. The loading content (LC) and encapsulation efficiency (EE) of TBO-CHI-PPS micelles were 5.2% and 52%, respectively. The critical micelle concentration (CMC) was found to be 0.012 mg/mL. The mechanism of thymol release from chitosan micelles involves micelle disassembly following ROS generation during irradiation. Positive micelles effectively bound to the negative surface of the biofilm, generating a large amount of ROS after irradiation, followed by micelle disassembly and thymol release. This mechanism allows for the controlled and targeted release of thymol, to effectively eliminate bacterial biofilms.

An earlier study by Zolfaghari et al. [42] also used toluidine blue O (TBO) as a light-activated antimicrobial photosensitizer for the elimination of *S. aureus*. Wang et al. confirmed the hydrophobic–hydrophilic nature of TBO-CHI-PPS caused by ROS generated during the oxidation of a hydrophobic thioether to a hydrophilic sulfoxide or sulfone, according to a study by Kim et al. [43]. The authors’ use of toluidine blue O (TBO) as an ROS generator (that can be activated by light) represents an innovative strategy for inducing antimicrobial activity in the biofilm environment. The studies conducted by Wang et al. demonstrated the generation of reactive oxygen species (ROS) by chitosan micelles under the influence of radiation, leading to the simultaneous release of thymol. This dual functionality, in which ROS trigger the release of thymol and exert additional bactericidal activity, provides a unique mechanism for combating bacterial biofilms.

## 5. Chitosan-Thymol Films Modified with Various Carriers/Compounds

Advances in wound care require biomaterials that can provide multifunctional benefits via antimicrobial activity to promote tissue regeneration. Chitosan and alginate, which are known for their biocompatibility and healing properties, have been extensively studied as biomaterials for wound healing. Alginate microparticles, on the other hand, are excellent carriers for encapsulating and controlling the release of therapeutic agents. By integrating thymol-loaded alginate microparticles with chitosan–gelatin matrices, it is possible to create a hybrid system that exploits the antimicrobial properties of thymol while benefiting from the structural and therapeutic properties of the composite film. In order to exploit the therapeutic potential of thymol in wound care, research was initiated in 2022 to produce chitosan–gelatin films integrated with thymol-loaded alginate microparticles (CS-GEL/Thymol-ALG MPs) using 2 mg of pure thymol in 1 mL of tween 80 [31]. CS-GEL/Thymol-ALG MPs were prepared by electrospraying and were incorporated into chitosan–gelatin films for controlled drug delivery and subsequent thymol release according to a pseudo-Fickian diffusion mechanism [31]. The encapsulation efficiency value for CS-GEL/Thymol-ALG MPs was set at 90.0% ± 0.3%. This result is much higher than most of the literature values. For example, Zhu et al. [44] evaluated the microencapsulation of thymol in poly(lactide-co-glycolide) using emulsion solvent evaporation and reported a maximum encapsulation efficiency of 47.19% + 1.99%. In addition, Maciel et al. [45] studied thymol-loaded zein microparticles prepared by spray-drying and obtained a 49.5% encapsulation efficiency. Ahmady et al. [report the loading capacity (LC) of thymol within the alginate microparticles as 8.6% ± 0.4%. Thymol release from the composite film is likely to occur via diffusion, with thymol molecules moving from an area of higher concentration (inside the film) to an area of lower concentration (outside the film). The controlled and prolonged release of thymol from the film allows for long-lasting antimicrobial and antioxidant effects, contributing to its effectiveness as a wound dressing. Owing to the combination of these components, the resulting composite film exhibited a controlled and prolonged release of thymol, overcoming the antimicrobial limitations of traditional chitosan–gelatin films. The antibacterial activity of CS-GEL/Thymol-ALG MPs against *S. aureus* and *E. coli* was evaluated and showed a significantly higher antibacterial activity after 24 h of contact, with percentage reductions of 99% and 50%, respectively.

The fabrication of a composite film containing thymol-loaded alginate microparticles in a chitosan–gelatin matrix represents a novel approach to developing advanced wound dressings. The encapsulation of thymol in alginate microparticles allowed for a controlled and prolonged release of thymol, increasing its therapeutic potential, and demonstrated antimicrobial activity, controlled release, and the promotion of wound healing, suggesting that such a composite film holds promise for meeting the multifaceted requirements of advanced wound care.

Poly(dimethylsiloxane) (PDMS), which is well known for its mechanical strength and flexibility, is of interest for biomaterial modifications. The interaction between biomaterials and their mechanical properties is crucial, particularly in applications such as wound dressings, where flexibility and strength are important. Integrating PDMS with existing biopolymer systems, such as chitosan–alginate films, offers the possibility of fine-tuning these mechanical properties while maintaining or even enhancing their therapeutic potential. Pires et al. [32] investigated the effect of PDMS on chitosan–alginate membranes infused with thymol and β-carotene, focusing on evaluating the mechanical properties, incorporation efficiency of bioactive compounds, and stability of PDMS chitosan–alginate film. Thymol (0.42 mg/mL in ethanol) was incorporated into chitosan–alginate polyelectrolyte complex (PEC) layers using various impregnation methods, including immersion in a solution and supercritical carbon dioxide impregnation/deposition. The loading efficiency of thymol in the PEC matrix was relatively low, not exceeding 0.28%, probably because of its high vapor pressure and solubility in supercritical carbon dioxide. Similar results were reported by Bombaldi Souza et al. [46], who observed a low incorporation of alpha-bisabolol into chitosan–guar gum membranes.

The study provided by Pires at al. focused on the effect of incorporating PDMS into chitosan–alginate films loaded with thymol and beta-carotene to improve the mechanical properties and overall performance of wound dressings, offering new insights into the use of PDMS in wound dressing materials. This approach provides new insights into the structural and functional aspects of the developed biomaterials.

The study conducted by Sharma et al. [33] investigated the encapsulation of thymol in a chitosan–aloe matrix to enhance its use in wound healing, addressing the limited bioavailability of thymol. The encapsulation process involved the integration of thymol into nanoemulsions and film dressings using a chitosan–aloe matrix. Homogenized *Aloe vera* gel was combined with chitosan solution in a 2:1 ratio (chitosan to *A. vera*). Thymol was dissolved in an 80% *v*/*v* aqueous ethanol solution, yielding concentrations ranging from 1 to 4 mg/mL. To prepare thymol-containing chitosan–aloe nanoemulsions, thymol solutions were introduced into a mixture of chitosan and *A. vera* at a ratio of 1:2. This mixture was subjected to continuous stirring at 50 °C for 4 h, followed by a 12 h period at an ambient temperature. The film dressing was prepared by casting the resulting nanoemulsions onto Petri dishes. The antimicrobial activity of the films was tested against pathogenic microorganisms, including *Bacillus*, *Staphylococcus*, *Escherichia*, *Pseudomonas*, *Klebsiella* and *Candida*, using the disk-diffusion method. The zone of inhibition was measured in centimeters. The results showed significant antimicrobial activity against all strains tested, with zones of inhibition ranging from 0.5 cm to 2.8 cm. By contrast, Rafieian et al. [47] obtained slightly different results. In their study, the antimicrobial activity of *A. vera* chitosan films was evaluated against *S. aureus* and *E. coli* using the colony-forming unit method. The results showed that chitosan films containing *A. vera* exhibited significant antimicrobial activity against *E. coli*, with the samples containing 50% *A. vera* being the most effective formulation. However, antimicrobial activity against *S. aureus* was not observed with the addition of *A. vera*, indicating that its presence in the system not only reduced the antimicrobial properties but also suppressed the inhibitory activity of chitosan against *S. aureus*. The encapsulation efficiency of thymol in the chitosan–*A. vera* matrix was reported to be 95.3% by Sharma et al. [33]. In addition, encapsulated thymol showed enhanced biological activity, as determined by DPPH assays, at 95.70%.

## 6. Optimizing the Synthesis of Thymol-Embedded Chitosan Nanoparticle

The precision and efficiency of drug delivery systems are crucial for maximizing their therapeutic benefits. Central to this endeavor is the design and optimization of encapsulation techniques tailored to specific bioactive compounds. In this context, much attention has been paid to targeted analyses aimed at tuning the manufacturing parameters of thymol-containing nanoparticles. To develop an effective thymol delivery system, Çakır et al. [34] initiated a study to optimize the production parameters of thymol-containing chitosan nanoparticles. Using a two-step emulsion–ion gelation process, the authors thoroughly investigated various factors affecting the process, including the temperature and concentrations of chitosan, thymol, Tween 80 and sodium tripolyphosphate (TPP). Using a factorial design method, the nanoencapsulation parameters were successfully optimized to obtain nanoparticles with the desired properties. The parameters that yielded the best results in terms of particle size, zeta potential, and encapsulation efficiency (EE) were: temperature—42 °C; chitosan concentration—3 mg/mL; thymol concentration—5.9 mg/mL; Tween 80 concentration—3 mg/mL; and TPP concentration—0.75 mg/mL. The EE of thymol-loaded chitosan nanoparticles was 66.4%. The authors noted that an increase in TPP enabled a higher EE, but the chitosan concentration was adversely affected. In addition, increasing the concentration of thymol from 3 to 6 mg/mL resulted in a higher EE. Similar effects have been reported by Woranuch et al. [48] and Keawchaoon et al. [49].

Çakır et al. used a two-step emulsion–ion gelation method to produce thymol-loaded chitosan nanoparticles; this is a novel aspect of the research. The approach allowed for the formation of nanoparticles under mild conditions without the use of high temperatures and organic solvents, which is beneficial for encapsulating bioactive compounds such as thymol.

## 7. Thymol-Induced Enhancement of Apoptotic Potential in Adenocarcinomic Human Alveolar Basal Epithelial Cells (A549)

Apoptosis, a regulated form of cell death, plays a key role in maintaining tissue homeostasis and is an important endpoint for assessing the therapeutic potential of novel compounds. The ability of bioactive compounds to modulate apoptotic pathways, particularly in specific cell lines, provides insight into their potential therapeutic applications. The A549 cell line, derived from human lung cancer cells, serves as a representative model for the study of lung diseases and related therapeutic interventions. Balan et al. [35] highlighted the enhanced apoptotic capacity of thymol in A549 cells when encapsulated in chitosan at a concentration of 20 mg/mL in DMSO. Thymol-loaded chitosan nanoparticles (thymol-NPs) were characterized by a size of 282.5 nm and an encapsulation efficiency of 74.08 ± 0.73%. This encapsulated form of thymol exhibited a pronounced apoptotic effect on A549 cells compared with its unencapsulated counterpart. The IC50 values for thymol and thymol-NPs in the cytotoxicity assay were determined to be 111.4 μg/mL and 99.57 μg/mL, respectively. For the 200 μg/mL thymol-NP, the viability of A549 cells was only 4.5%, which is significantly better than using thymol alone, which showed 19% viability at the same volume. In addition, other studies of the compounds encapsulated in chitosan indicated better cytotoxicity [50,51]. Balan et al. [35] showed for the first time that thymol-NPs are more potent than free thymol in inducing apoptosis in A549 cells. That is, A549 cells treated with thymol showed 2.25% early apoptotic cells and 90.36% late apoptotic cells. The population of early and late apoptotic cells in thymol-NP-treated A549 cells increased to 2.35 and 93.74%, respectively. Their research confirmed the results of earlier studies, in which thymol induced apoptosis in many types of cancer cells [52]. The in vivo toxicity studies in Swiss albino mice showed that the thymol-NP did not cause any toxic symptoms, death, or gross pathological results up to a concentration of 1000 mg/kg. The LD50 value of thymol was observed as 980 mg/kg body weight in rats. In the sub-acute toxicity test, the animals treated with 1000 mg/kg of thymol-NP for 28 days did not show any significant changes in behavior, body weight, organ weight, or organ histology.

Balan et al. showed that chitosan nanoparticles containing thymol exhibited enhanced cytotoxicity and apoptotic activity against A549 lung cancer cells compared with free thymol. In vivo toxicity studies in mice have demonstrated the safety of thymol-containing chitosan nanoparticles at specific concentrations. These results suggest the potential for further development of thymol-NPs as safe and potent drug candidates for cancer treatment.

## 8. Advancing Bone Regeneration through Thymol-Loaded Polymeric Hydrogels

Bone regeneration remains a key challenge in orthopedic and tissue engineering, requiring the development of innovative biomaterials to promote and accelerate the healing process. Hydrogels have been used in this area. Jurczak et al. [53] presented hydrogels for scaffolds in the engineering and regeneration of bone and cartilage tissues to provide mechanical support and promote cell growth and regeneration. The use of hydrogels as platforms for the delivery of bioactive factors allows for the controlled release of growth factors, thereby increasing their therapeutic potential. The authors concluded that hydrogels are promising alternatives to traditional bone grafts, which are associated with surgical risks and high costs. However, further research is required to optimize the properties and performance of hydrogels as scaffolds, including the development of their mechanical strength, biocompatibility, and ability to promote tissue regeneration. Guillén-Carvajal et al. [54] used chitosan, collagen, and gelatin as biopolymers in hydrogel scaffolds for bone-tissue engineering. Their research showed promising results in terms of biocompatibility, degradation control, and the mechanical properties of such combinations. The crosslinking of the polymer network, chemical properties of the polymer network, interaction between the polymer precursors and fillers, and addition of inorganic components had significant effects on the physical parameters, drug release rate, and matrix crosslinking in the hydrogels. The addition of TiO_2_ nanoparticles to the hydrogels improved their thermal stabilities. The hydrogels showed potential to promote osteoblast proliferation, accelerate bone repair, increase the compressive modulus, and allow cell infiltration, tissue ingrowth, nutrient transport, and drug diffusion. Ghandforoushan et al. [55] stated in their article that hydrogels hold promise for bone regeneration as they can be used as scaffolds for stem cells injected via needles to replace tissue defects. Injectable hydrogels combined with stem cells can be used as stem cell carriers to repair tissue defects and minimize invasiveness. Such hydrogels have high water-retention capacity and mimic the moist environment of native cells. Injectable hydrogels are characterized by properties such as biocompatibility, low cytotoxicity, shear-thinning properties, and controlled biological and physicochemical properties.

The integration of thymol into polymeric hydrogel matrices offers a new approach to exploit its therapeutic potential for bone tissue regeneration. However, the effect of thymol on osteogenesis has not yet been thoroughly investigated. In a study conducted by Lavanya et al. [36], the authors successfully synthesized a drug delivery vehicle using semi-interpenetrating polymer network (SIPN) hydrogels consisting of sodium alginate and poly(2-ethyl-2-oxazoline) (SA/Pox) loaded with different concentrations of thymol (100, 150, and 200 μM). In addition, these hydrogels were coated with chitosan (CS) to enhance their bioactivity and achieve a sustained and prolonged release of thymol. The release percentages at day 25 were: 59.3 ± 2.8%, 71.3 ± 1.4%, and 78.1 ± 1.7% for SA/Pox/CS-Thy containing 100 μM, 150 μM and 200 μM thymol, respectively. The synthesis of CS-coated SIPN (SA/Pox/CS-Thy) hydrogels with thymol involves both ionic gelation and polyelectrolyte complexation techniques. The addition of chitosan to hydrogels significantly improved their physicochemical and material properties. In addition, the hydrogels showed compatibility with mouse mesenchymal stem cells (mMSCs). After culturing mMSCs on the hydrogels, thymol stimulated osteoblastic differentiation, as evidenced by the presence of calcium deposits at the cellular level. The expression of RUNX2, a transcription factor critical for bone formation, and other biomarkers of differentiation was significantly increased in mMSCs cultured on SA/Pox/CS-Thy hydrogels. In particular, thymol present in the SA/Pox/CS-Thy hydrogels significantly activated the TGF-β/BMP signaling pathway, which is known to play an important role in osteogenesis. In a rat tibial bone-defect model, the incorporation of thymol into SA/Pox/CS hydrogels resulted in enhanced bone regeneration. The long-term release of thymol from the SA/Pox/CS hydrogels effectively promoted osteoblast differentiation in vitro and bone formation in vivo. An in vitro cytocompatibility assessment proved no significant changes in observed cell metabolic activity, membrane integrity, or morphology. Equally beneficial activity in bone regeneration was reported by Ressler [56], who stated that different characterization methods are used; therefore, the results of different studies cannot be properly compared, and no definitive conclusions can be drawn about the application potential of such materials.

A study conducted by Lavanya et al. comprehensively evaluated these hydrogels, including their physicochemical properties, cytocompatibility, drug release kinetics, and in vivo performance in a rat tibial bone-defect model. This comprehensive approach provides valuable insights into potential bone regeneration. The study showed that thymol released from the hydrogels played a key role in activating TGF-β/BMP signaling pathways and components that promote osteoblast differentiation. This insight into how thymol promotes osteogenesis is the novelty of the present study.

## 9. Chitosan/Thymol Materials for Oral Administration/Periodontal Treatment

Chitosan/thymol materials represent a novel and promising approach in dentistry, particularly for oral administration and periodontal treatment, combining the mucoadhesive properties of chitosan with the antibacterial and anti-inflammatory effects of thymol. Such biomaterials respond to the growing demand for more effective, biocompatible, and non-toxic therapeutic options in dentistry. These materials can offer a dual action: improving oral health by combating pathogens and reducing inflammation while promoting tissue regeneration and healing.

Kardestani et al. [37] investigated the antibacterial effect of a thymol–chitosan nanocomposite (TLCN) on *Actinomyces viscosus* and *Streptococcus mutans*, which are the main caries-forming bacteria associated with tooth decay. The authors compared chlorhexidine and its combination in their study but did not mention the amount of thymol used in the analysis. The best MIC was related to TLCN+chlorhexidine 2.66 μg/mL for both tested bacteria. The nanocomposite itself had a worse MIC (7.3 ± 1.15 μg/mL; 6.6 ± 1.154 μg/mL) than chlohexidine alone (5.33 ± 2.4 μg/mL; 4.66 ± 1.15 μg/mL). A study by Zhu et al. [44] also showed the promising antimicrobial activity of microencapsulating thymol in poly (lactide-co-glycolide) (PLGA) against *E. coli* and *S. aureus*. The mechanism of action of TLCN in the inhibition of these pathogens has not yet been clearly defined. It can be inferred that the antimicrobial action of TLCN can be attributed to the properties of chitosan and thymol. The combination of chitosan and thymol in the TLCN may enhance their antimicrobial activity through synergistic interactions. Chitosan may increase the solubility and stability of thymol, allowing for better delivery and release. Chitosan nanoparticles can also improve the penetration of thymol into bacterial cells, thereby enhancing their antimicrobial activity. The combination of chitosan and thymol may have a cumulative effect, targeting different sites or mechanisms of bacterial growth and survival, which requires further investigation.

A study by Kardestani et al. demonstrated the potential of TLCN as an effective antimicrobial agent against these bacteria. An evaluation of the cytotoxicity of TLCNs on cancer cells (KB) and normal cells (HGF1-PI1) showed selective toxicity against cancer cells and non-toxicity to normal cells, indicating the potential of TLCN as a targeted therapy for oral cancer.

Chittratan et al. [38] evaluated the antimicrobial properties of chitosan-coated thymol (CST) and used it as a capping agent for the synthesis of gold nanoparticles (AuNPs) against oral carious bacteria. UV-visible spectrometry was used to confirm the successful synthesis of CST and AuNPs. X-ray diffraction (XRD) was performed to analyze the crystallographic structures of the nanoparticles. Transmission electron microscopy (TEM) was used to photograph the morphology, particle size, and distribution of AuNPs. Elemental zeta potential analysis was used to measure the zeta potential of the nanoparticles. ^1^H NMR spectroscopy was used to confirm the successful synthesis of CST and AuNPs. The authors used 79.32 mg of pure methanol dissolved in 1 mL of dimethylformamide. The study showed that the chitosan-coated gold nanoparticles (CST-AuNPs) exhibited good antimicrobial activity against pathogenic bacteria including *S. mutans* and *S. sobrinus,* as demonstrated by agar well diffusion assay. The thymol modification of CST-AuNPs increased their antimicrobial activity, with distinct zones of inhibition of 15.90 and 14.25 mm, respectively. The minimum inhibitory concentration (MIC) and minimum bactericidal concentration (MBC) of CST-AuNPs against *S. mutans* were 25 and 100 mg/L, respectively. For *S. sobrinus*, the MIC and MBC values were 100 and 200 mg/L, respectively. The synthesis described by the authors using the Mannich reaction and the application of z represents a novel approach for obtaining new materials with potential antimicrobial properties.

The study conducted by Alvarez Echazú et al. [39] in 2021 compared the potential of unmodified and dodecenylsuccinic anhydride (DDSA)-modified chitosan hydrogels for sustained thymol release and to evaluate their properties and performance in a rat model of ligature-induced periodontitis. Various methods have been used to assess the properties and performance of modified gels, such as the characterization of chitosan (CHI) and DDSA–chitosan hydrogel by FTIR, ^13^C NMR, and rheological measurements; the ultrastructure assessment of the hydrogels by SEM; in vitro tests to determine antimicrobial and antioxidant activity; and cytotoxicity tests using mouse fibroblast cells to compare biocompatibility. Thymol incorporation was 0.35 ± 0.03 mg for CHI and 0.33 ± 0.03 mg for DDSA-CHI per hydrogel. The study showed that thymol-loaded chitosan hydrogels exhibited antimicrobial activity against *S. aureus* and *P. aeruginosa* for two days. The CFU/mL decreased by seven log units for *S. aureus*, whereas for *P. aeruginosa,* the number of colony-forming units decreased from ~10^8^ to ~10^7^. Cytotoxicity studies of the DDSA-CHI hydrogel showed approximately 40% lower cell viability than unmodified CHI. Thymol-loaded DDSA-CHI presented free radical scavenging activity for 5 days and was the highest on the third day, with 10% inhibition. The thymol-loaded hydrogels showed antimicrobial activity against *S. aureus* and *P. aeruginosa*, as well as antioxidant activity. The combination of antimicrobial and antioxidant properties in a localized drug delivery system represents a new approach to the multifactorial nature of periodontal diseases. The evaluation of hydrogels in a rat model of periodontitis provides valuable insights into their potential therapeutic effects in a biological context.

A qualitative and quantitative summary of the presented research results on the biological activity and formulation characterization of TLCBS is presented in Table 2.

## 10. Thymol Biotransformation

**Figure 3:**
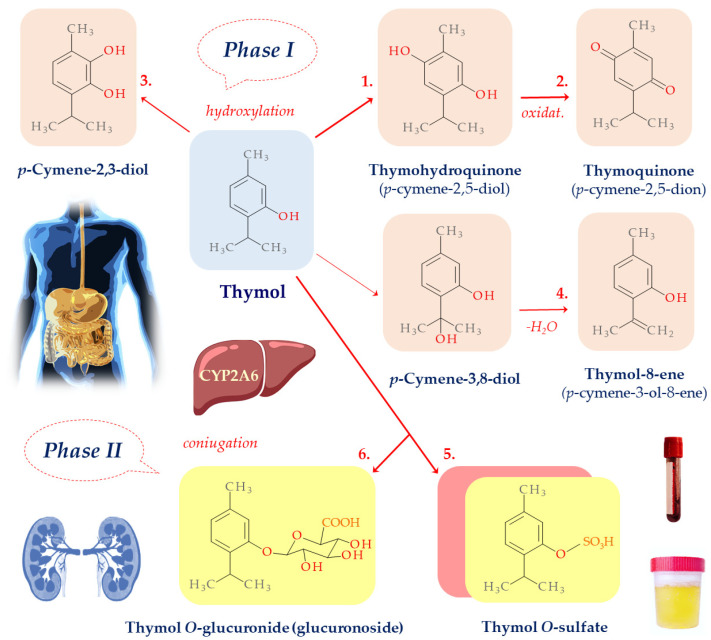
Thymol metabolism in humans.

In chitosan systems with thymol as the component, its biotransformation plays a key role, offering insight into how it interacts with and is modified by biological systems. The absorption, distribution, metabolism, and excretion (ADME) of thymol in healthy volunteers have been studied in vitro and in vivo. As a phenolic derivative, thymol is subjected to phase I and II metabolism in living organisms. Studies on thymol bioavailability have been published since the 1930s; however, scientific data on its ADME in humans are still incomplete. Dong et al. [57], in model studies with human recombinant CYP isoforms, found that thymol undergoes phase I metabolism, most likely involving three cytochrome isoforms—CYP1A2, CYP2A6, and CYP2B6—that can catalyze the hydroxylation of the aromatic ring and side chain of thymol (Figure 3). The incubation of human liver microsomes in the presence of NADPH resulted in the formation of a single major metabolite of thymol. The involvement of CYP2A6 in thymol metabolism was confirmed using 8-methoxy psoralen, a selective inhibitor of this cytochrome isoform. CYP2A6 is expressed not only in the human liver but also in other tissues such as the nasal epithelium, trachea, lungs, and esophagus. However, owing to the high inter-individual variability of CYP2A6, different pharmacokinetic and clinical responses can be predicted in humans following thymol administration [57,58].

The chemical structures of thymol metabolites were elucidated in healthy volunteers after oral administration of a single dose of thymol (50 mg). Metabolites were analyzed in the urine samples using GC-MS. Phase I metabolism in humans leads to hydroxylation of the aromatic ring and, to a lesser extent, of the isopropyl side chain. This resulted in more polar derivatives with an additional -OH group substituted at the *para* or *ortho* position relative to the hydroxyl group in thymol structure (Figure 1). The *para* isomer thymohydroquinone (*p*-cymene-2,5-diol) (1) was predominant, whereas the *ortho* isomer *p*-cymene-2,3-diol (3) was detected in much smaller amounts after a longer extraction time from the urine sample. Thymohydroquinone then undergoes oxidation to thymoquinone (*p*-cymene-2,5-dione) (2), which is the main product of the phase I metabolism. The hydroxylation of the isopropyl group leads to the formation of an unstable *p*-cymene-3,8-diol and the corresponding dehydration product (−H_2_O) thymol-8-ene (*p*-cymene-3-ol-8-ene) (4), which, unlike its precursor, is also detected in human urine [58]. The occurrence of thymohydroquinone among thymol metabolites in the urine animal models and humans has also been reported previously [59,60].

A clinical study involving a small group of healthy volunteers was conducted to determine the systemic availability and pharmacokinetics of thymol after its oral administration in humans [61]. However, no free thymol (unbound) was detected in either the plasma or urine. Phase II metabolites such as thymol *O*-sulfate (5) and *O*-glucuronide (6) conjugates were detected in the urine by LC-MS. Thymol *O*-sulfate, but not thymol *O*-glucuronide, was also detected in the plasma at a maximum after 2 h. The average elimination half-life was estimated to be 10.2 h. The amount of thymol conjugates excreted in urine within 24 h was 16.2% ± 4.5% of the ingested dose. The renal clearance of thymol was 0.27 L/h, suggesting high binding to proteins and reabsorption in the kidneys; the volume of distribution was 14.7 L. These data indicate that thymol *O*-sulfate remained mainly in the extracellular compartment. Moreover, despite the lack of free thymol in the plasma, thymol *O*-glucuronide is eliminated by the kidneys, similar to *O*-sulfate. The ratio of these two phase II metabolites is likely dose-dependent. A higher dose can shift metabolism toward *O*-glucuronide. Kohlert et al. [62] suggested that thymol *O*-sulfate might be reabsorbed in the proximal tubule after glomerular filtration. This process could be preceded by *O*-sulfate cleavage and thymol release under the activity of aryl sulfatases or by brush border enzymes, followed by reabsorption. However, in vitro studies on phenolic compounds have suggested that glucuronidation and sulfation can also occur in enterocytes. Abid at al. [63] demonstrated that in the microsomal fraction of Caco-2 cells, the production of thymol *O*-glucuronide might begin in the intestine. Thymol has also been excreted in the urine as sulfate and glucuronide conjugates in rabbits, dogs and rats [60,64].

Only a few studies have been conducted on thymol metabolism in the human intestine. As shown in a study by Mosele et al. [65], thymol is poorly metabolized by the gut microbiota during model fermentation in vitro (using human feces as the inoculum). None of the gut microbiota metabolites were quantified in human feces (except for one participant) after a 3-week intake of thyme phenolics (including thymol ~25 mg/kg of preparation). These data confirmed previous observations that thymol is efficiently absorbed during its passage into the small intestine. Thyme phenols were efficiently absorbed in the upper part of the gastrointestinal tract, and consequently, only unabsorbed negligible residues were observed in the fecal samples. Thymol remained stable during fermentation in vitro, possibly because of its persistent chemical structure (a phenol derivative), which may impede its microbial degradation. However, it should be taken into account that thymol has direct antimicrobial properties.

## 11. Thymol Antimicrobial Activity and Cell Membrane Interactions

The bacterial membrane, a key structure in prokaryotic organisms, serves as a selective barrier and a functional platform for various cellular processes. Composed mainly of lipid compounds, including phosphatidic proteins with minimal sugar content, this membrane facilitates the regulated transfer of chemicals into and out of the bacterial cells. The integrity and functionality of this membrane are crucial for maintaining the cytoplasmic environment and the viability of bacterial organelles. Thymol can interact with bacteria through its ability to alter cell membranes or affect processes inside the bacterial cells.

Nourbakhsh et al. [66] elucidated the mechanism by which thymol interacts with and penetrates the bacterial membranes. According to the authors, this process is characterized by the affinity of thymol for the lipid bilayer, where it integrates with the hydrophobic region and induces changes in the membrane fluidity and permeability. Such changes threaten the integrity of the membrane, leading to the leakage of important cellular components, culminating in the death of bacterial cells. Furthermore, the effect of thymol on the fatty acid composition of the membrane contributes to its antimicrobial efficacy by disrupting the membrane permeability and structural integrity. Sharma et al. [67] showed that thymol can spontaneously penetrate the bacterial membrane and localize to regions adjacent to the lipid core groups. This was attributed to the weak amphiphilic nature of thymol. Complementing these findings, Wu et al. [68] demonstrated the potent antimicrobial activity of thymol, particularly against *Aeromonas hydrophila*. The antimicrobial activity of thymol was also associated with the disruption of cell membrane structures, which led to the exudation of intracellular material, resulting in bacterial cell death.

Although this appears to be the main mechanism by which thymol interacts with the bacterial cell membrane, other studies have indicated that it may also interact with its internal targets.

The growth of *S. aureus* was effectively inhibited by thymol, as confirmed by Li et al. [69]. In this case, thymol can depolarize the membrane potential of *S. aureus*, leading to its damage and cell death. To understand the mechanism of the antimicrobial action, the concentrations of soluble NADP^+^/NADPH and ATP in the cytoplasm were determined. At a concentration of 300 μg/mL, thymol caused an increase in NADP^+^ and a decrease in the cytoplasmic levels of NADPH and ATP. This suggests a possible leakage of intracellular components and a disruption of the physiological NADP^+^/NADPH balance. Other experiments and the authors’ results suggest that cell membrane lipid oxidation levels were significantly increased by thymol. In turn, the catalytic enzyme NOX2 can regulate the NADP^+^/NADPH balance; therefore, NOX2 may be a potential target of thymol in cells. In this study, thymol showed the best antimicrobial activity, with MICs ranging from 125 µg/mL for pathogens such as *Acinetobacter baumannii*, *Pasteurella aerogenes*, and *Salmonella typhimurium* to 250 µg/mL for *Bacillus subtilis*, *Klebsiella aerogenes*, *Klebsiella pneumoniae*, *Serratia marcescens*, *S. aureus* and *Streptococcus agalactiae*. In a study by Marchese et al. [70], thymol was found to inhibit the growth of *S. aureus* at various concentrations. Studies have shown that the minimum inhibitory concentrations (MICs) of thymol against *S. aureus* are 0.31 mg/mL, 0.156 mg/mL and 62.5 μg/mL. In addition, the concentration of thymol required to disrupt the membrane integrity of *S. aureus* is 750 mg/mL. These findings indicated that thymol exhibits antimicrobial activity against this pathogen at relatively low concentrations, suggesting its potential as an effective antimicrobial agent.

The comparison of the antimicrobial activity of pure thymol with that of systems incorporating thymol has been the subject of many studies. The antimicrobial activity of thymol incorporated into the structure of a dry zeolite composite (ZEO4A/thymol) was compared to that of pure thymol against four microbial pathogens: *C. albicans*, *E. coli*, *P. aeruginosa* and *S. aureus* [71]. Thymol inhibited the growth of these strains to varying degrees. *S. aureus* was the most sensitive strain, whereas *P. aeruginosa* was the most resistant. The minimum inhibitory concentration (MIC) of thymol was set at 2.5 mM for *C. albicans* and *E. coli*, while the MIC for *S. aureus* was 1.25 mM. The viability of the strains was completely suppressed by a thymol concentration of 75 mM, except for *P. aeruginosa*, which showed some survival. The authors concluded that the ZEO4A/thymol composite showed enhanced antibacterial activity against *E. coli* and *S. aureus* compared with pure thymol. The composite was found to have higher thymol content and better physical stability, leading to increased antimicrobial activity. However, it should be noted that *P. aeruginosa* showed resistance to both thymol and the composite. Also, a Piri-Gharagei et al. [28] study showed that thymol-loaded chitosan nanogel (Ty-CsNG) had higher inhibitory activity against both Gram-negative and Gram-positive multidrug-resistant bacteria compared to free thymol (Ty) alone. A time-kill test at different concentrations of the chitosan nanogel and chitosan nanogel loaded with thymol showed that Ty-CsNG had higher antimicrobial activity than free thymol after 24 h. In addition, they found that the antibiofilm activity of Ty-CsNG was significant compared to free chitosan nanogel and free thymol.

Elshamy et al. [72] found that a chitosan-based film containing a nanoemulsion of thyme oil showed significant antimicrobial activity against both Gram-negative (*Escherichia coli* spp.) and Gram-positive (*Bacillus subtilis* spp.) bacteria. The film showed inhibition zone diameters ranging from 11.75 to 14.7 mm and 16.75 to 17.70 mm against *Bacillus subtilis* and *E. coli* spp. respectively. The minimum inhibitory concentration (MIC) of thyme oil in the chitosan-based film was estimated at 0.1 mg/mL, which was lower than the MIC of thyme oil extracted from *Thymus vulgaris* spp. (2 mg/mL). Moghtaderi et al. [73] used a methacryloyl gel (GelMa)-based encapsulation of thymol in a nanoniosome, highlighting the enhanced antimicrobial efficacy against Gram-positive and Gram-negative pathogens compared to free thymol, and demonstrated a significant reduction in biofilms with this formulation. The average MIC values of thymol, niosomal thymol and the final encapsulation product were 125, 15.62–31.25 and 1.95–3.9 µg/mL against Gram-positive strains (*S. aureus* and *B. subtilis*) and 250–500, 62.5 and 7.81–15.62 µg/mL against Gram-negative strains (*K. pneumonia* and *P. aeruginosa*), respectively. The above reports clearly indicate the increased antimicrobial activity of thymol incorporated into different systems compared to that of pure thymol.

## 12. Limitations of Presented Studies and Future Directions

While promising, these presented studies have several limitations that need to be addressed in future research and clinical implementations, and most studies have been limited to in vitro conditions that do not fully replicate the complex biological environments of living organisms. Upcoming research should include in vivo models to better understand the interactions and efficacy of nanoparticles in biological systems. The current research focuses on the efficacy of nanoparticles against a narrow range of bacteria, limiting their broader antimicrobial applications. Therefore, it is necessary to expand the spectrum of antimicrobial activity to a wider range of pathogens. Potential interactions between nanoparticles and other oral microorganisms remain largely unexplored. These interactions are needed to assess the overall impact on the efficacy and safety of nanoparticles in oral health applications, and extensive human studies are needed to translate laboratory findings into clinical practice. Future studies should also evaluate the safety, efficacy and optimal dosage of nanoparticle formulations in humans. There is a lack of data on the long-term stability, scalability and manufacturing process of these nanoparticles; therefore, the focus should be on these aspects to ensure their practical application. Studies on the long-term effects and potential toxicity of nanoparticle formulations are lacking. It is critical to understand the long-term safety implications, especially for applications involving prolonged or repeated exposure. Comparative studies with existing commercially available wound dressings and medication removal systems are lacking. Therefore, further investigations should include comparative studies to determine the advantages and benefits of systems based on these nanoparticles.

## 13. Conclusions

This review of several medical applications of thymol–chitosan systems highlights their multifaceted potential in the medical field. These nanogels have demonstrated significant antimicrobial and antibiofilm activity compared to pure thymol against multidrug-resistant strains, making them promising candidates for combating difficult bacterial infections. The encapsulation of thymol in innovative chitosan nanogel formulations not only proves their antimicrobial properties but also increases cellular uptake and apoptotic efficacy while reducing the required active concentration, making them promising for targeted anticancer therapy. Some research gaps, such as the lack of more extensive in vivo studies and comparative analyses with existing formulations and technological aspects, also set new directions for future research in this field and realizing the full potential of the innovative therapeutic properties of these nanoformulations.

## Figures and Tables

**Table 1:** General summary of research topics and results on TLCBS.

	TLCBS	Synopsis	Main Findings	Refs.
1.	Thymol-loaded chitosan nanogels	Nanogels for delivering thymol	- Effective against multidrug-resistant bacterial strains - Reduction in biofilm formation - Negligible cytotoxicity	[28]
2.	Tragacanth gum and chitosan as a nanocarrier for thymol	Bipolymer nanocarriers to enhance thymol’s therapeutic properties	- Two-phase release kinetics - Enhanced anti-inflammatory and antioxidant properties	[29]
3.	Light-controlled chitosan micelles	Light-driven chitosan micelles for thymol delivery	- Generation of ROS upon irradiation - Effective against bacterial biofilms	[30]
4.	Chitosan–gelatin films with thymol-loaded alginate microparticles	Production of chitosan–gelatin films with thymol for wound care	- Controlled and prolonged thymol release - Effective antimicrobial activity - Accelerated wound healing	[31]
5.	Impact of PDMS on chitosan–alginate films	Influence of PDMS on chitosan–alginate films with thymol	- Sustained thymol release - Optimal tensile strength	[32]
6.	Encapsulation of thymol within a chitosan–*Aloe vera* matrix	Enhance applicability in wound healing	- Encapsulation promoting the prevention of bacterial infection - Encapsulated thymol had higher biological activity	[33]
7.	Fine-tuning production of thymol-loaded chitosan nanoparticles	Optimization of production parameters for thymol-containing nanoparticles	- Efficient encapsulation of thymol - Improved solubility and bioavailability	[34]
8.	Apoptotic potential enhancement in A549 cells	Study of thymol’s effect on A549 cells when encapsulated in chitosan	- Pronounced apoptotic effect - Safe drug candidate for non-small cell lung cancer	[35]
9.	Bone regeneration through thymol-loaded polymeric hydrogels	Study of thymol’s role in bone tissue regeneration	- Promotion of osteoblast differentiation - Enhanced bone regeneration in vivo	[36]
10.	Antibacterial effect of thymol–chitosan nanocomposite	Impact on tooth decay	- The combination of chitosan and thymol may have a cumulative effect on teeth decay	[37]
11.	Antimicrobial properties of chitosan-coated thymol	Enhancing antimicrobial properties against oral caries bacteria.	- The potential to control bacterial growth in the oral cavity is emphasized	[38]
12.	Chitosan hydrogels for extended release of thymol with dodecenylsuccinic anhydride	Potential of hydrogels for prolonged thymol release and their properties in a model of periodontitis	- Chitosan hydrogels with thymol have a more favorable effect on periodontal tissue adhesion and better potential for treating periodontitis	[39]

**Table 2:** Qualitative and quantitative summary of the biological activity and formulation characterization of TLCBS.

	Authors	Thymol Concentration	Methods of Analysis	Results
1.	Piri-Gharaghie et al., 2022 [28]	0.25 mg/mL in ethanol	1. Antibacterial and antibiofilm activities against *Acinetobacter baumannii*, *P. aeruginosa*, *S. aureus* 2. Assessment of the antibiofilm effect 3. Stability and encapsulation efficiency assay	1. Ty-CsNG had the highest inhibitory effect against pathogensMIC for *Acinetobacter baumannii* 32–128 μg/mL; *S. aureus* 8–64 μg/mL; *P. aeruginosa* 2–32 μg/mL 2. Expression of biofilm-related genes ompA and pgaB were significantly down-regulated by Ty-CsNG 3. Ty-CsNG exhibited stability for up to 60 days at 4 °C, with an average size of 82.71 ± 9.6 nm and encapsulation efficiency of 76.54 ± 0.62%
2.	Sheorain et al., 2019 [29]	4 mg/mL in ethanol	1. Preparation of nanoformulation using ionic complexation method 2. In vitro anti-inflammatory activity using HRBC stabilization method 3. In vitro antioxidant activity using DPPH assay	1. The concentration of chitosan influenced the particle size and encapsulation efficiency of the thymol nanoformulation the best results—200 mg tragacanth gum and 400 mg chitosan concentration, encapsulation efficiency 98.72% with a ratio of 1:2 (tragacanth gum:chitosan) 2. In vitro anti-inflammatory activity—89. 60% membrane stabilization 3. In vitro antioxidant activity—to 90% at a thymol concentration of 0.5 mg/mL in formulation
3.	Wang et al., 2019 [30]	1 mg/mL in DMSO	1. Antibacterial activities evaluation *S. aureus* and *P. aeruginosa* 2. Determination of thymol loading content (LC) and encapsulation efficiency EE 3. Determination of critical micelle concentration (CMC)	1. The MIC and MBC values of TBO-CHI-PPS micelles against *S. aureus* were found to be 165 μg/mL and 330 μg/mL, respectively, against *P. aeruginosa* 330 μg/mL and 660 μg/mL, respectively 2. The LC and EE of TBO-CHI-PPS micelles were 5.2% and 52%, respectively 3. The CMC of TBO-CHI-PPS micelles 0.012 mg/mL
4.	Ahmady et al., 2022 [31]	2 mg/mL in tween 80	1. Antibacterial activities evaluation *S. aureus* and *E. coli* 2. Determination of thymol loading content (LC) and encapsulation efficiency (EE)	1. The antibacterial activity CS-GEL/Thymol-ALG MPs showed a significantly higher antibacterial activity after 24 h contact, with percentage reductions of 99% and 50%, respectively 2. The EE nad LC of CS-GEL/Thymol-ALG MPs were 90.00 ± 0.3% and 8.6% ± 0.4%, respectively
5.	Pires et al., 2018 [32]	0.42 mg/mL in ethanol	1. Ionic gelation technique wasemployed for the formulation of thymol-loaded CS nanoparticles 2. Incorporation efficiency of thymol and β-carotene 3. Hemolytic properties and thrombus formationon the ASTM E96-90D	1. The addition of poly(dimethylsiloxane) (PDMS) to the chitosan–alginate films improved their mechanical properties, increasing their Young’s modulus and tensile strength. 2. Thy loading efficiency did not exceed 0.28% 3. The addition of PDMS to the chitosan–alginate films significantly increased their thrombogenicity compared to the control
6.	Sharma et al., 2023 [33]	1 mg/mL to 4 mg/mL in aqueous ethanol (80% *v*/*v*)	1. Antimicrobial activity testing *Bacillus*, *Staphylococcus*, *Escherichia*, *Pseudomonas*, *Klebsiella*, *Candida* using Kirby–Bauer disc diffusion method 2. UV spectrophotometry: for the DPPH radical scavenging assay 3. Encapsulation efficiency (EE)	1. Significant antimicrobial activity, with zones of inhibition ranging from 0.5 cm to 2.8 cm 2. Encapsulated thymol showed a bioactivity of 95.70% in DPPH assays 3. EE at 95.3%
7.	Çakır et al., 2020 [34]	3 or 6 mg/mL in Tween 80	Encapsulation efficiency (EE)	66.4% EE for thymol-loaded chitosan nanoparticles
8.	Balan et al., 2022 [35]	20 mg/mL in DMSO	1. Encapsulation efficiency and drug loading capacity 2. Cytotoxicity using the MTT assay and Annexin V/FITC staining followed by flow cytometry analysis 3. Sub-acute toxicity testing on Swiss albino mice	1. Encapsulation efficiency 74.08 ± 0.73%. and drug loading capacity 37.0 ± 0.3% 2. The IC_50_ values for thymol and thymol-NP in cytotoxicity assay were 111.4 μg/mL and 99.57 μg/mL, respectively 3. In the sub-acute toxicity test, the animals treated with 1000 mg/kg of thymol-NP for 28 days did not show any significant changes
9.	Lavanya et al., 2023 [36]	100, 150, 200 μM in 1 mL DMSO	1. In vivo studies using a rat tibial bone-defect model 2. Drug release analysis using UV spectrophotometric 3. In vitro cytocompatibility assessment and cell morphology evaluation and osteoblast differentiation at the cellular and molecular levels	1. The hydrogels promote bone regeneration in a rat tibial bone-defect model, with the SA/Pox/CS-Thy hydrogels showing the highest bone mineral density and bone volume fraction 2. Thymol release percentages at day 25 were 59.3 ± 2.8%, 71.3 ± 1.4% and 78.1 ± 1.7% for SA/Pox/CS-Thy containing 100 μM, 150 μM and 200 μM thymol, respectively 3. No significant changes in cell metabolic activity, membrane integrity, or morphology observed in vitro cytocompatibility assessment
10.	Kordestani et al., 2023 [37]	not provided	1. Minimum inhibitory concentration (MIC) activity against *Streptococcus mutans* and Actinomyces viscosus by microdilution in broth 2. The viability assay of cell lines of tumor cells (KB) and normal cells (HGF1-PI1) with different concentrations of the nanocomposite for 72 h at 37 °C with 5% CO2, and then the 50% cytotoxic concentration (CC50) was determined	1. The TLCN + chlorhexidine nanocomposite showed the lowest minimum bactericidal concentration (MBC) at 2.66 μg/mL for both bacteria 2. The exposure of the tested bacteria to TLCN dose-dependently increased the protein leakage, particularly at 1/2 and 1/3 MIC, markedly elevating the protein leakage in the tested bacteria. The 50% cytotoxic concentration (CC50) of nanocomposite against normal (HGF1-PI1) and cancer (KB) cells were 149.6 and 68.4 μg/mL, respectively
11.	Chittratan et al., 2022 [38]	79.32 mg/mL indimethylformamide	1. Mannich reaction to graft thymol onto the chitosan side chain to synthesize chitosan-grafted thymol (CST) 2. Agar well diffusion assay for the antimicrobial activity against *S. mutans* and *S.* sobrinus. Minimum inhibitory concentration (MIC) and minimum bactericidal concentration (MBC) assay	1. The Mannich reaction led to the synthesis of chitosan-grafted thymol (CST). The degree of substitution of CST was determined to be 10.0% by nuclear magnetic resonance (NMR) spectroscopy 2. MICs and MBCs for CST-AuNPs against *S. mutans* were 25 mg/L and 100 mg/L, respectively, and *for S. sobrinus* 100 mg/L and 200 mg/L, respectively
12.	Alvarez Echazú et al., 2017 [39]	0.35 ± 0.03 mg forCHI and 0.33 ± 0.03 mg for DDSA-CHI per hydrogel.	1. Plate count method for antimicrobial activity of the hydrogels against *S. aureus* and *P. aeruginosa*. 2. Cytotoxicity of DDSA-CHI 3. Antioxidant activity using DPPH method 4. Thymol incorporation and release using Folin–Ciocalteau method	1. The hydrogels were effective against *S. aureus*, with a seven log-unit decrease in colony forming units (CFU/mL) observed. For *P. aeruginosa*, the CFU/mL decreased from ~10^8^ to ~10^7^ 2. Cytotoxicity studies: The DDSA-CHI hydrogel presented a ca. 40% lower cell viability when compared to unmodified CHI 3. Antioxidant studies indicate potential as an antioxidant delivery system with more 10% inhibition 4. The thymol-loaded DDSA-CHI hydrogel showed sustained release of thymol over 24 h, with a cumulative release of 60%

## Data Availability

Not applicable.
